# Analgesia strategy for inguinal hernia repair in children: a systematic review and network meta-analysis of randomized clinical trials based on regional blocks

**DOI:** 10.3389/fped.2024.1417265

**Published:** 2024-08-02

**Authors:** Xing Xue, Yuxin Zhou, Na Yu, Zhihua Yang

**Affiliations:** ^1^ Department of Anesthesiology, Jinshan Branch of Shanghai Sixth People’s Hospital, Shanghai, China; ^2^ The First School of Clinical Medicine, Lanzhou University, Lanzhou, China

**Keywords:** inguinal hernia repair, pediatrics, analgesia, regional blocks, network meta-analysis

## Abstract

**Background and objective:**

Despite its acknowledged benefits, the selection of an optimal regional block for analgesia pediatric hernia surgery remains a subject of debate. This study endeavored to conduct a network meta-analysis and systematic review of randomized clinical trials, aiming to amalgamate insights from both direct and indirect comparisons concerning the analgesic effectiveness and safety of various regional blocks post-inguinal hernia repair in children.

**Method:**

A comprehensive literature search was performed across PubMed, EMBASE, Web of Science, and the Cochrane Library up to 12 November 2022 by two independent reviewers, employing a standardized protocol. The inclusion criteria encompassed randomized trials focusing on children undergoing inguinal hernia repair utilizing either local infiltration analgesia or regional analgesia. The primary outcomes assessed were pain scores at 2, 6, and 24 h post-operation.

**Results:**

The initial search yielded 281 records relating to 1,137 patients. The analysis of ranking probability indicated that Paravertebral Block (PVB) holds the highest likelihood (88% and 48%) of being the most effective in alleviating pain at 2 h and 6 h post-surgery. Trans vs. Abdominis Plane Block (TAPB) emerged as the superior choice for mitigating pain (83%) and decreasing morphine consumption (93%) at 24 h following the operation. Local Anesthetic Infiltration (LAI) was identified as the most effective in shortening the hospital stay, with a 90% probability.

**Conclusions:**

Regional anesthesia significantly enhances postoperative pain management in pediatric inguinal hernia repair surgery. For short-term postoperative pain relief, PVB emerges as the most effective technique. Meanwhile, TAPB provides more prolonged analgesia. Although TAPB does not exhibit a pronounced advantage in short-term analgesia, its simplicity and the absence of a need for a special position render it a viable option. However, the interpretation of these results should be approached with caution due to the presence of limited data and heterogeneity.

**Systematic Review Registration:**

PROSPERO (CRD42022376435; www.crd.york.ac.uk/prospero).

## 1 Introduction

Children frequently endure considerable postoperative pain and discomfort following inguinal hernia repair surgery (1). Despite advances in minimally invasive techniques for this procedure in recent years, postoperative pain continues to significantly contribute to discomfort and hinder the swift return to normal activities for pediatric patients (2). Regional blocks are heralded as both safe and effective for enhancing pediatric surgical outcomes and reducing the incidence of opioid-related complications (3–5). In addition to caudal analgesia (CA) (5) and local anesthetic infiltration (LAI) (6), the use of peripheral nerve blocks, such as the Ilioinguinal-Iliohypogastric block (II/IHB) (7), Transvs. Abdominis Plane Block (TAPB) (8) Retrolaminar Block (RLB) (9), Paravertebral Block (PVB) (10) and Quadratus Lumborum Block (QLB) (11) has expanded due to their lower risk of side effects. However, the best approach for managing postoperative pain in pediatric inguinal hernia repair remains contested. So, the aim of this network meta-analysis (NMA) and systematic review was to collate and assess the data from direct and indirect comparisons regarding the efficacy and safety of various regional blocks for postoperative pain management.

## 2 Methods

### 2.1 Study design

This study's protocol was prospectively registered with PROSPERO (CRD42022376435; www.crd.york.ac.uk/prospero), and our reporting adheres strictly to the PRISMA-NMA checklist for systematic reviews and NMAs (12)**.**

### 2.2 Search strategy and selection criteria

We systematically searched PubMed, EMBASE, Web of Science, and the Cochrane Library from their inception until 12 November 2022, conducted by two independent researchers. Our search strategy employed a broad range of free-text keywords and subject headings pertinent to analgesia following pediatric inguinal hernia repair. Specific search terms used for PubMed are detailed in Supplementary File. The selection of eligible trials was finalized through consensus, with any disputes resolved by a third party. Inclusion criteria included (1) pediatric patients undergoing inguinal hernia repair; (2) interventions involving local infiltration analgesia or regional analgesia; and (3) the study's design as a randomized controlled trial (RCT). Exclusion criteria encompassed (1) non-English literature and (2) studies without accessible data.

### 2.3 Outcome measures

The primary outcome assessed in this study was pain scores at 2, 6, and 24 h post-surgery. Secondary outcomes included the consumption of rescue analgesia drugs at 2, 6, and 24 h after surgery, the length of hospital stay, and the incidence of postoperative complications such as nausea, vomiting, and urinary retention.

Data collection and analysis were methodically executed by two researchers who independently screened the articles by title, abstract, and full text. Data compilation was carried out using Microsoft Excel, with any discrepancies or disagreements resolved by a third researcher.

### 2.4 Risk of bias and quality assessment

The potential risk of bias in the included studies was evaluated using the Cochrane risk of bias tool (13). This assessment was independently conducted by two reviewers, focusing on aspects such as random sequence generation, allocation concealment, blinding, completeness of outcome data, selective reporting, and other potential sources of bias. Each criterion was classified as “Yes,” “No,” or “Unclear,” indicating a high, low, or unclear risk of bias, respectively. Any disagreements between reviewers were resolved through consultation with a third researcher.

### 2.5 Statistical methods

The data was analyzed using a random-effects Bayesian framework for Network Meta-Analysis, executed through ADDIS software (Groningen, the Netherlands, www.drugis.org). Network plots were generated using STATA 15.0 software (Stata Corporation, College Station, TX, USA), and the quality of included studies was assessed with RevMan 5.3 (Cochrane Community, London, England). The mean difference (MD) with 95% confidence intervals (CIs) served as the measure of outcome. Data were analyzed using random-effects and consistency models within the network meta-analysis. The Markov chain Monte Carlo (MCMC) method was employed for estimation, with four chains run using non-informative priors. Convergence was assessed by the potential scale reduction factor (PSRF), where a value close to 1 indicated satisfactory convergence. Node splitting analysis was used to evaluate the consistency between direct and indirect comparisons. *P *≥ 0.05 indicated that no significant inconsistency was found, and consistency models were used to estimate the results. In contrast, the inconsistency model was selected (14). Comparisons were also ranked based on their outcomes.

## 3 Results

### 3.1 Study selection and characteristics

**Figure 1:**
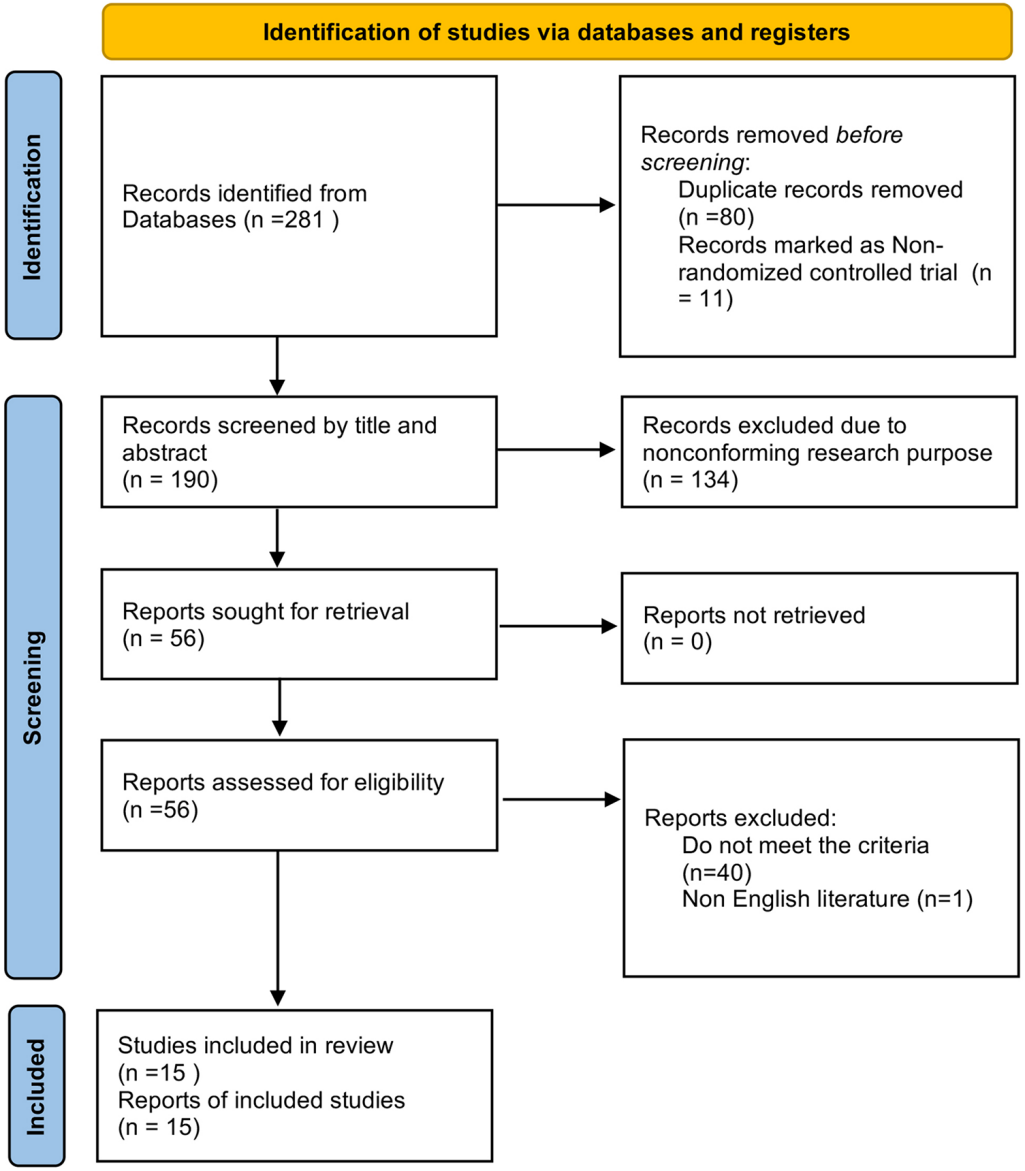
Flow diagram of the study selection process.

**Figure 2:**
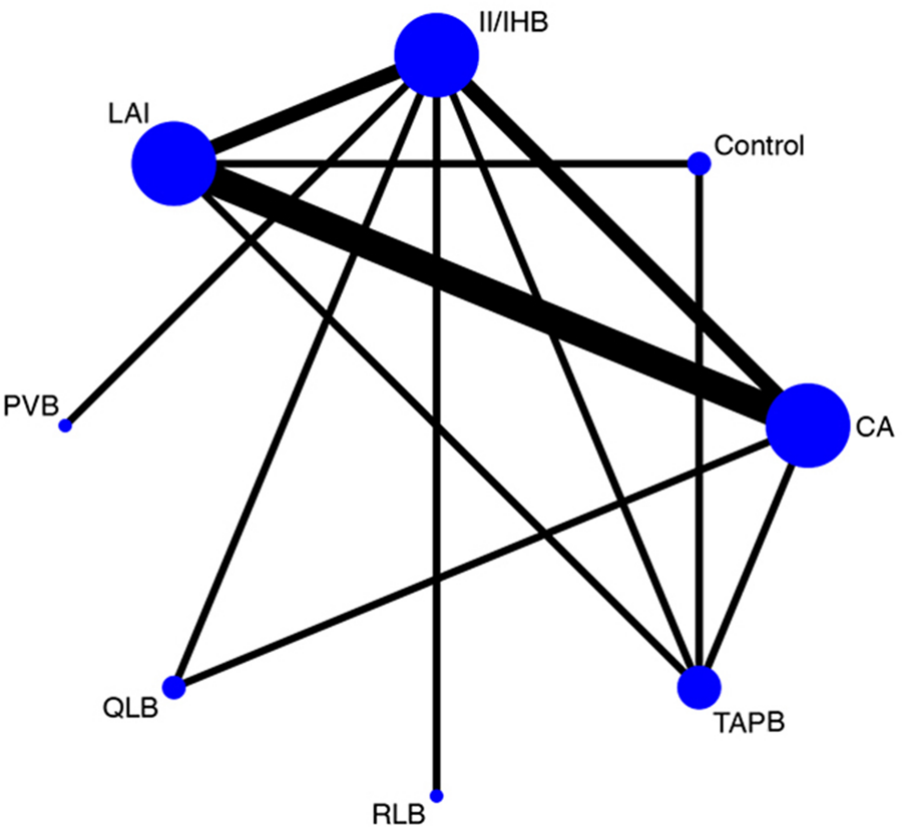
Network plot for the all interventions. Circles represent the intervention as a node in the network, lines represent direct comparisons using randomized controlled trials and the thickness of lines corresponds to the number of randomized controlled trials included in each comparison.

From the initial online database searches, we retrieved 281 records related to 1,137 patients. Following a detailed review process, 15 randomized controlled trials were identified as meeting our inclusion criteria (3, 6–10, 15–23). Figure 1 depicts the flowchart outlining the steps of the literature search and study selection. Among these 15 studies, fourteen featured comparisons between two different interventions (or intervention vs. control), and one study evaluated a single intervention arm. The comparisons across these studies were varied, including one study comparing LAI to a control group (6); and another comparing TAPB to control (17). Four studies assessed CA against LAI (15, 18, 21, 22), while two studies examined CA vs. II/IHB (7, 15). Additional comparisons included CA vs. TAPB (8), CA vs. QLB (23), II/IHB vs. RLB (9), II/IHB vs. LAI (16), I/IHB vs. PVB (10), II/IH vs. QLB (19), LAI vs. TAPB (3), and II/IHB vs. TAPB (20). The detailed characteristics of these studies are summarized in Table 1, providing a comprehensive overview of the interventions evaluated, the patient populations studied, and the outcomes measured. Figure 2 showcases the network plot.

**Table 1:** The characteristics of the selected included studies.

Author/year	Country	Age (year)	Group (*n*)	Main mode of anesthesia	Local anesthetic for block	Outcomes
Toker (15)	Turkiye	4.08 ± 1.61	CA(25)	GA	0.5 ml/kg 0.25% bupivacaine	[1] [2]
	Turkiye	4.08 ± 1.61	II/IHB(25)	GA	0.3 ml/kg 0.25% bupivacaine	[1] [2]
	Turkiye	4.08 ± 1.61	LAI(25)	GA	0.2 ml/kg 0.5% bupivacaine	[1] [2]
Sahin (3)	Turkiye	4.50 ± 1.50	TAPB(29)	GA	0.5 ml/kg 0.25% levobupivacaine	[2] [4]
	Turkiye	4.5 ± 1.5	LAI (28)	GA	0.2 ml/kg 0.25% levobupivacaine	[2] [4]
ŠŠujica (6)	Serbia	3.4 ± 1.9	LAI(50)	GA	0.5 mg/kg 0.5% levobupivacaine	[2]
	Serbia	3.5 ± 1.9	Control(50)	GA	saline	[2]
Casey (16)	USA	5.67 ± 2.58	LAI(30)	GA	0.25 ml/kg 0.25% bupivacaine	[3]
	USA	5.75 ± 2.91	II/IHB(30)	GA	0.25 ml/kg 0.25% bupivacaine	[3]
Abdelbaser (17)	Egypt	2 ± 2.6	TAPB(20)	GA	0.4 ml/kg 0.25% bupivacaine	[2]
	Egypt	1.54 ± 2.96	Control(20)	GA	saline	[2]
Kumar (8)	India	4.09 ± 2.12	TAPB(56)	GA	0.5 ml/kg 0.2% ropivacaine	[2] [4]
	India	3.73 ± 1.78	CA(56)	GA	1 ml/kg 0.2% ropivacaine	[2] [4]
Naja (10)	Lebanon	7.3 ± 1.9	II/IHB(40)	GA	0.3 ml/kg of the mixture^a^	[1] [4]
	Lebanon	7.4 ± 1.8	PVB(40)	GA	0.3 ml/kg of the mixture^a^	[1] [4]
Desai (7)	India	3.28 ± 2.06	CA(55)	GA	0.75 ml/kg 0.25% bupivacaine	[1] [2] [3]
	India	3.88 ± 1.77	II/IHB(45)	GA	0.3 ml/kg 0.25% bupivacaine	[1] [2] [3]
Splinter (18)	Canada	4.6 ± 2.5	CA(96)	GA	1 ml/kg 0.2% bupivacaine	[1] [3] [4]
	Canada	4.9 ± 2.7	LAI (104)	GA	0.3 ml/kg bupivacaine 0.25%	[1] [3] [4]
Samerchua (19)	Thailand	2.4 ± 1.6	QLB(19)	GA	0.5 ml/kg 0.25% bupivacaine	[2] [4]
	Thailand	3.2 ± 2.6	II/IHB(19)	GA	0.2 ml/kg 0.25% bupivacaine	[2] [4]
Alseoudy (9)	Egypt	3.15 ± 1.48	RLB(30)	GA	0.5 ml/kg 0.25% bupivacaine	[1]
	Egypt	3.4 ± 0.7	II/IHB(30)	GA	0.5 ml/kg 0.25% bupivacaine	[1]
Fredrickson (20)	New Zealand	3.9 ± 2.7	TAPB(21)	GA	0.3 ml/kg of a 50: 50 mixture of lidocaine 1% and ropivacaine 1% with epinephrine	[1]
	New Zealand	4.4 ± 3.7	II/IHB(20)	GA	0.3 ml/kg of a 50: 50 mixture of lidocaine 1% and ropivacaine 1% with epinephrine	[1]
Machotta (22)	Germany	1.07 ± 1.23	LAI(28)	GA	0.2 ml/kg 0.5% bupivacaine	[1]
	Germany	0.46 ± 1.67	CA(30)	GA	1 ml/kg 0.25% bupivacaine	[1]
Öksüz (23)	Turkey	3.92 ± 0.36	QLB(27)	GA	0.7 ml/kg 0.25% bupivacaine	[1] [4]
	Turkey	3.7 ± 0.35	CA(25)	GA	0.7 ml/kg 0.25% bupivacaine	[1] [4]
Schindler (21)	Australian	4.0 ± 1.9 3.0 ± 3.1	CA(27) LAI(27)	GA GA	0.7 ml/kg of 0.25% bupivacaine 0.7 ml/kg of 0.25% bupivacaine	[1] [4] [1] [4]

Data are presented as mean ± SD. GA, genera anaesthesial; CA, caudal analgesia; QLB, quadratus lumborum block; TAPB, transversus abdominis plane block; PVB, paravertebral blockade; II/IHB, ilioinguinal/Iliohy- pogastric nerve block; RLB, retrolaminar block; LIA, local infiltration analgesia; NA, not available.

Outcomes: [1] Pain score; [2] Rescue analgesia drug consumption; [3] Hospital stay; [4] Postoperative complications.

^a^ lidocaine 2% 6.5 ml, bupivacaine 0.5% 6 ml, fentanyl 50 ug ml^−1^ and clonidine 150 ug m^−1^.

### 3.2 Quality assessment

**Figure 3:**
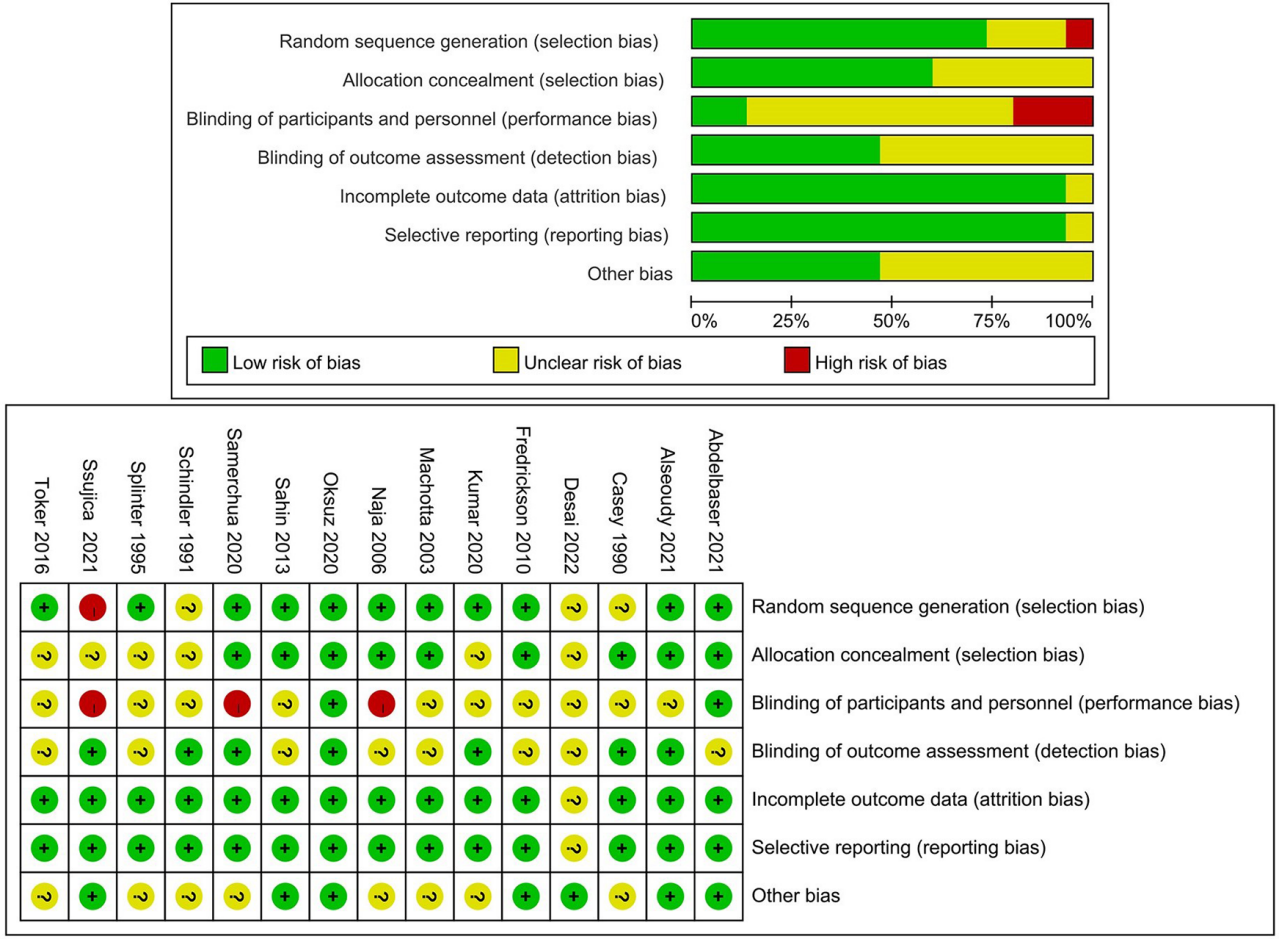
Cochrane collaboration risk of bias graph and summary. Green circle, low risk of bias; red circle, high risk of bias; yellow circle, unclear risk of bias.

The assessment of study quality and risk of bias for the included randomized controlled trials (RCTs) was conducted using the Cochrane risk of bias tool. This thorough evaluation revealed that the overall quality of the RCTs incorporated into our network meta-analysis ranged from high to moderate. Specifically, in terms of randomized sequence generation, a crucial element for ensuring the validity of trial outcomes, eleven of the studies adequately described their method for generating randomized sequences. However, three studies did not provide details regarding their randomization process. Regarding allocation concealment, another key factor that prevents selection bias, nine studies adequately reported their methods to ensure the concealment of allocation sequences from both researchers and participants. Blinding of outcome assessment, essential for mitigating detection bias, was explicitly mentioned in only two of the included studies. Figure 3 provides a visual summary of the risk of bias assessment.

### 3.3 Primary outcomes

**Figure 4:**
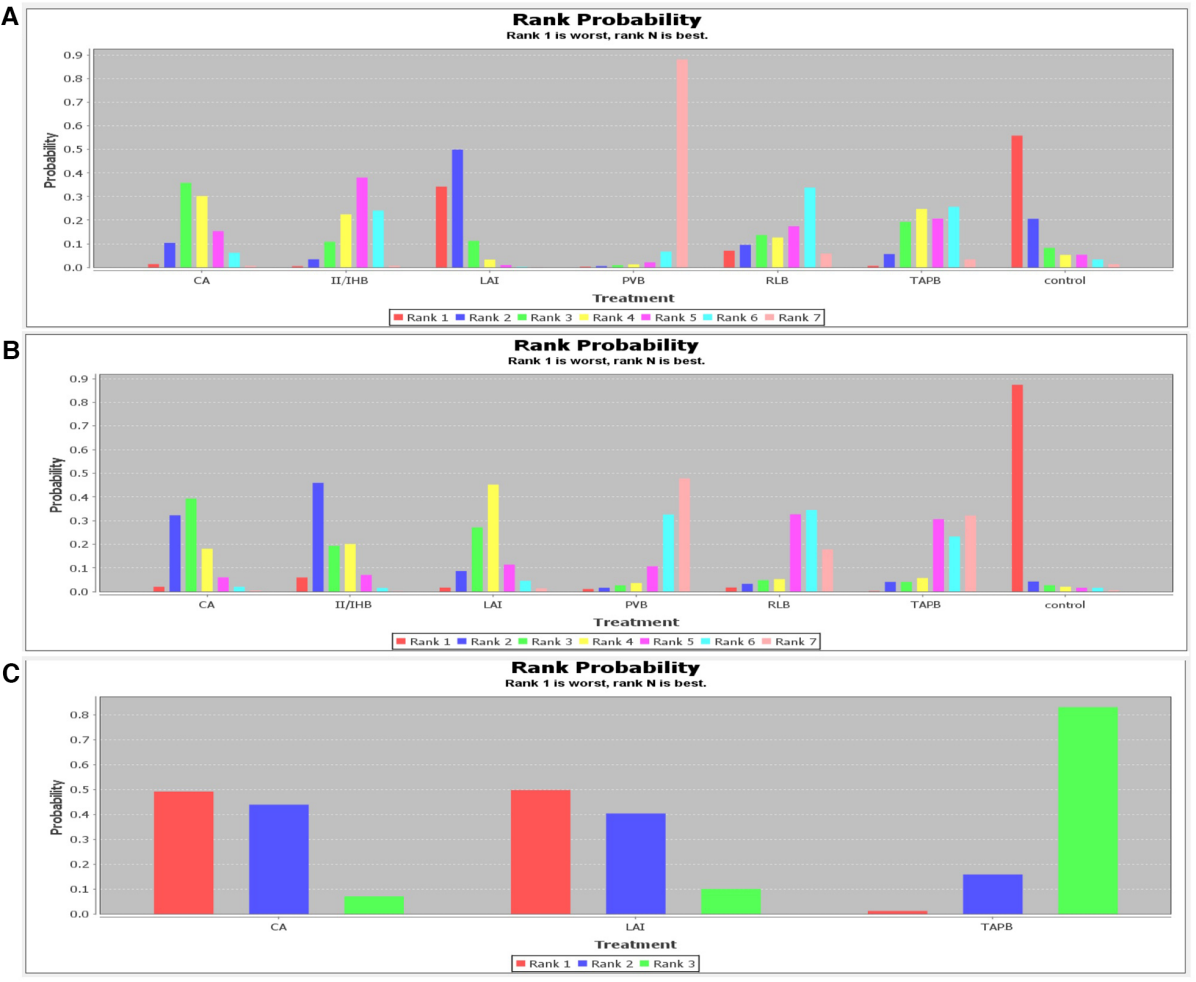
Rank probability of rest pain scores. (**A**) 2 h; (**B**) 6 h; (**C**) 24 h.

Pain Scores at 2 Hours Post-Surgery: Eleven studies provided data on pain scores at 2 h after surgery. The interventions evaluated included CA, II/IHB, LAI, PVB, RLB, TAPB, and a control group. The network analysis indicated that CA, II/IH, LAI, PVB, RLB, and TAPB were all superior to the control group in managing pain. The ranking probability diagram identified PVB as the most effective method (88%) for alleviating pain at 2 h post-surgery (Figure 4 and Supplementary Table S1).

Pain Scores at 6 Hours Post-Surgery: Seven studies reported on pain scores at 6 h, involving the same interventions as above. Analysis showed that all listed interventions were more effective than the control group. PVB was identified as the most favorable option (48%) for reducing pain at 6 h post-surgery (Figure 4 and Supplementary Table S2).

Pain Scores at 24 Hours Post-Surgery: Two studies provided data for pain scores at 24 h, involving CA, LAI, and TAPB. TAPB was highlighted as the most effective (83%) in preventing pain 24 h post-surgery (Figure 4 and Supplementary Table S3).

### 3.4 Secondary outcome

#### 3.4.1 Rescue analgesia drug consumption

**Figure 5:**
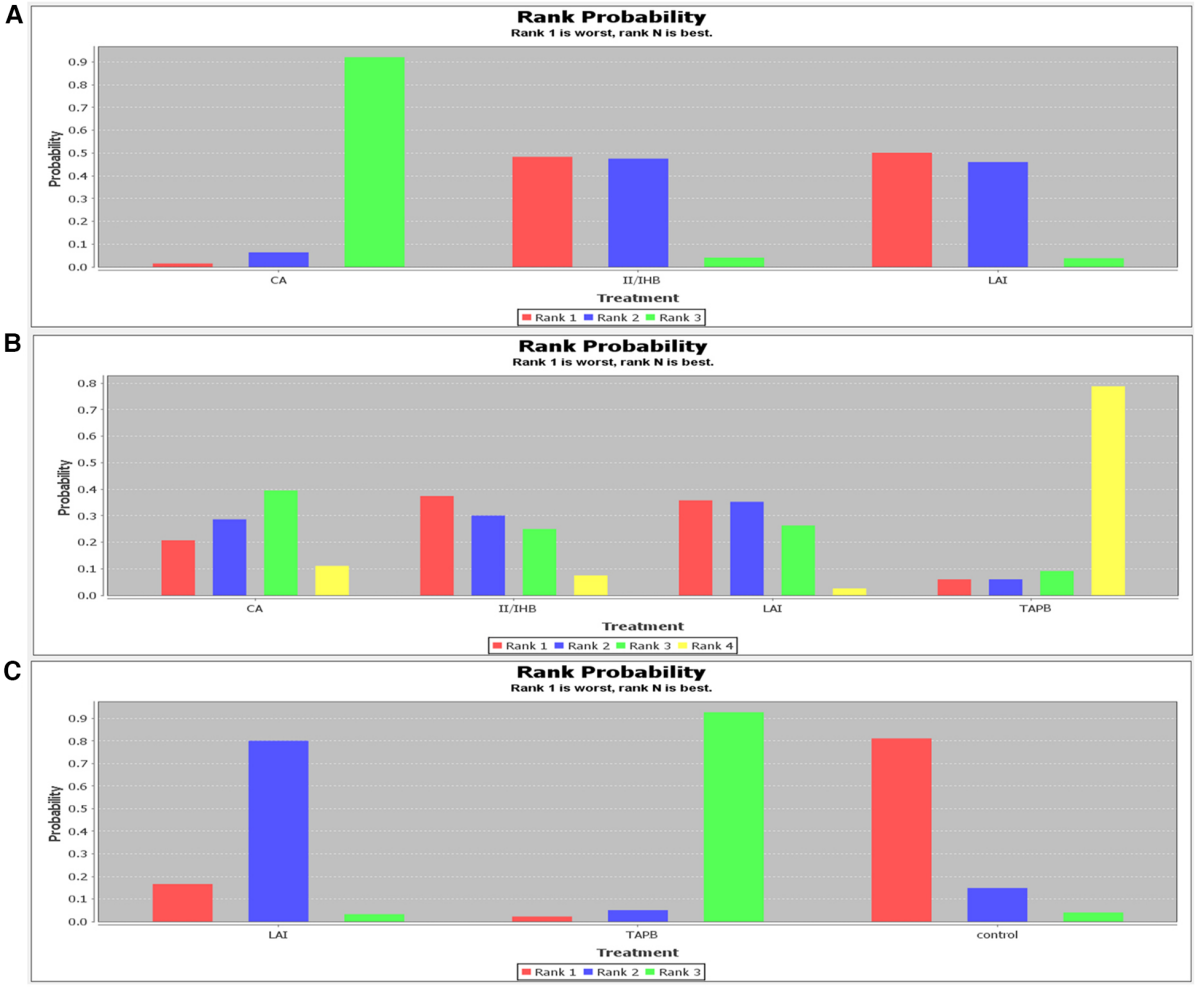
Rank probability of rescue analgesia drug consumption. (**A**) 2 h; (**B**) 6 h; (**C**) 24 h.

At 2 h post-surgery, only one study reported on the consumption of rescue analgesia, involving CA, II/IHB, and LAI. CA was found to be the most effective (92%) in reducing the need for additional analgesia at 2 h post-surgery (Figure 5 and Supplementary Table S4).

At 6 h, two studies reported on rescue analgesia consumption with interventions including CA, II/IHB, LAI, and TAPB. TAPB emerged as the most effective (79%) in reducing morphine consumption at 6 h post-surgery (Figure 5 and Supplementary Table S5).

At 24 h, two studies provided data on rescue analgesia consumption involving LAI, TAPB, and a control group. TAPB was shown to be the most effective (93%) in decreasing morphine consumption at 24 h post-surgery (Figure 5 and Supplementary Table S6).

#### 3.4.2 Length of hospital stay

**Figure 6:**
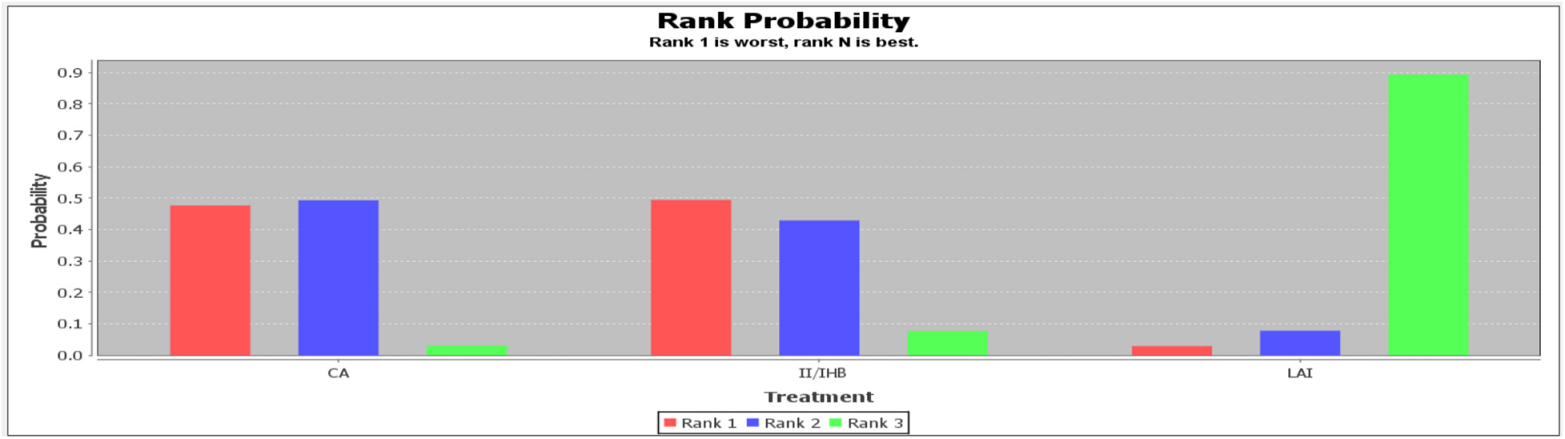
Rank probability of length of hospital stay.

Three studies reported on the length of hospital stays, comparing the effectiveness of CA, I/IHB, and LAI. According to the ranking probability diagram, LAI was identified as the most effective option, with a 90% probability of reducing hospital stays the most significantly (Figure 6 and Supplementary Table S7).

#### 3.4.3 Postoperative complications

**Figure 7:**
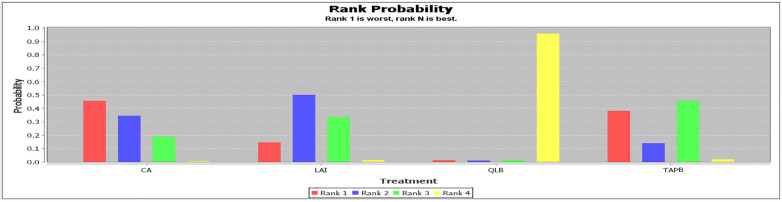
Rank probability of PONV.

Seven studies provided data on the incidence of postoperative complications. Of these, five studies reported occurrences of nausea and vomiting (3, 8, 18, 21, 23), involving interventions such as CA, LAI, QLB, and TAPB. The QLB was highlighted as the most effective method, with a 96% probability, for reducing the incidence of PONV (Figure 7 and Supplementary Table S8). Additionally, one study documented cases of transient femoral nerve blocks [10], and two studies reported urinary retention (18, 21).

## 4 Discussion

This NMA demonstrated that PVB emerged as the most effective technique for providing perioperative analgesia at 2 and 6 h post-inguinal hernia repair in children. TAPB was identified as the optimal choice for analgesia 24 h after surgery. Regarding the consumption of rescue analgesia drugs, CA was the most effective at reducing the need for additional pain relief at 2 h, whereas TAPB proved best at both 6 and 24 h. In terms of facilitating patient recovery, LAI was found to be the most effective in shortening the length of hospital stays. The most commonly reported side effects included PONV, with urinary retention and transient femoral nerve blocks also noted.

Inguinal hernia repair is a common surgical procedure in pediatric patients, which often accompanied by considerable postoperative pain and discomfort. Effective pain management is of significant importance for the rapid recovery of children following hernia repair. Laparoscopic approaches, as a minimally invasive surgery, may be an effective measure to reduce postoperative discomfort and provide earlier return to normal activities. However, study showed that postoperative pain was still an important cause of discomfort in children despite minimally invasive surgery was performed (2). Consequently, a multitude of analgesic approaches have been explored, with opioids emerging as the most frequently utilized class of analgesics. However, the concerns surrounding opioid side effects and addiction, coupled with the challenges in adequately assessing and treating pain in pediatric populations, have underscored the importance of non-opioid analgesics and multi-modal analgesia strategies. In recent years, regional analgesia has gained attention as a safe and effective technique that can enhance pediatric surgical outcomes and reduce opioid-related complications such as nausea, vomiting, itching, and respiratory depression, particularly in inguinal hernia repairs (11, 24, 25).

A range of regional anesthetic techniques is available to ensure effective postoperative pain management following pediatric inguinal hernia repair. Despite this variety, the determination of the most effective method remains a subject of debate among researchers and clinicians (3, 7, 9, 23).

CA has been a popular choice for postoperative analgesia in children undergoing lower abdominal surgery (26). However, it has been associated with several adverse effects, including urine retention, accidental dural puncture, retroperitoneal hematoma, bowel perforation, and the risk of systemic toxicity (27, 28).

Machotta et al. (22) suggested that local anesthesia infused into the surgical wound could provide comparable postoperative pain relief to CA, with a lower incidence of side effects. The advent of ultrasound-guided regional anesthesia has established peripheral nerve blocks as effective alternatives to the caudal block for postoperative pain control in children undergoing inguinal herniotomy. Ultrasound-guided II/IH and TAPB are particularly favored for their efficacy in alleviating postoperative pain (29–31). Recent meta-analyses have shown that II-IHB, TAPB, and CA offer comparable postoperative analgesia. Compared to CA, both II-IHB and TAPB significantly reduced postoperative motor blockade and hastened the time to urinate (32). Our NMA found no significant difference in pain relief between these techniques at 2, 6, and 24 h post-operation, which aligns with the findings of earlier studies (32). Although II-IHB may offer superior pain relief compared to TAPB, challenges in ultrasound image quality and procedural difficulty were noted (20). TAPB, accessible via various approaches, may influence its effectiveness (33, 34).

PVB involves the administration of local anesthetic adjacent to the thoracic vertebrae, specifically targeting the area around the spinal nerves as they emerge from the intervertebral foramina. This technique is relevant for inguinal hernia repairs due to the sensory innervation of the inguinal region by the ilioinguinal, iliohypogastric, and genitofemoral nerves. Notably, there is significant variation in the sensory innervation among individuals (35), and the hernia sac receives visceral innervation from spinal nerve roots T12-L2 (36), which abdominal wall blocks cannot address. PVB, however, can block the visceral innervation (10). Studies have shown that PVB, compared to the ilioinguinal nerve block, not only enhances and prolongs postoperative analgesia but also results in higher satisfaction among parents and surgeons (10). Moreover, PVB offers improved postoperative analgesic effects and reduces the need for postoperative analgesics while maintaining better hemodynamic stability during surgery compared to the II/IHB (10).

RLB serves as an alternative to PVB. In RLB, anesthetic is injected between the vertebral lamina and the deep paraspinal muscles, a technique first described by Pfeiffer et al. (37). RLB has been reported as an effective alternative to PVB [40] and can be performed either through palpation of anatomical landmarks or under ultrasound guidance (38). Given the absence of major vessels or nerves along the needle pathway, RLB is considered anatomically safe. Studies indicate that RLB offers superior postoperative analgesia compared to the ilioinguinal nerve block in children undergoing unilateral inguinal herniotomy (9). This NMA found no significant differences in pain control between these methods at 6 and 24 h. However, at 2 h post-operation, PVB was superior to LAI, suggesting PVB's effectiveness in short-term pain management. Regarding the consumption of rescue analgesia drugs, CA was the most effective at 2 h, while TAPB excelled at 6 and 24 h. LAI was identified as the most efficacious in reducing hospital stays.

QLB is a newer abdominal truncal block technique that provides analgesia for both the upper and lower abdomen. It involves the diffusion of local anesthetic between the posterior aspect of the quadratus muscle and the inner layer of the thoracolumbar fascia, which is located near the paravertebral space (39). QLB has proven more effective than traditional caudal block in multimodal analgesia settings (23) and has shown superior pain relief compared to the TAPB without adverse reactions (39).

Nevertheless, this NMA has several limitations. Firstly, the studies varied in their use of local anesthetics, with some employing levobupivacaine, ropivacaine, bupivacaine, or mixtures thereof. Secondly, the concentration and volume of the anesthetics differed, potentially influencing analgesic outcomes. Thirdly, adjuncts such as epinephrine, fentanyl, and clonidine were used in some trials. Fourthly, assessing pain in children is complex, influenced by factors like cognitive level, language ability, and cultural background, with various evaluation methods used across studies, such as FLACC (6, 7, 9, 17, 23), CHEOPS (3, 8, 18, 19, 21, 22) and VAS (10). Consequently, predicting efficacy and risk is challenging due to developmental differences among children of different ages. Sixth, only three studies (9, 17, 19) explicitly reported the use of open surgical and other 12 studies did not mention it. This maybe influence our finding because minimally invasive surgery had a certain effect on postoperative pain. Lastly, some outcomes were reported by a limited number of trials, and some treatments were unique to single studies, further complicating the analysis.

## 5 Conclusions

In conclusion, according to our NMA, regional anesthesia significantly enhances postoperative pain management in pediatric inguinal hernia repair surgery. For short-term postoperative pain relief, PVB emerges as the most effective technique. Meanwhile, TAPB provides more prolonged analgesia, making it the preferred choice for managing pain at 24 h post-surgery. Additionally, TAPB stands out for reducing the need for supplementary analgesia at both 6 and 24 h post-operation. While TAPB may not offer the best short-term analgesia, its simplicity and the fact that it does not necessitate a special position-being performable in the supine position-make it a practical option. Nonetheless, given the scarcity of data, particularly regarding newer blocking techniques, our conclusions should be interpreted with caution. There is a clear need for further research, including direct comparisons of different regional anesthesia techniques in the context of pediatric inguinal hernia repair, to solidify these findings.

## Data Availability

The original contributions presented in the study are included in the article/supplementary material, further inquiries can be directed to the corresponding author/s.
