# Supplementary material for: Analgesia strategy for inguinal hernia repair in children: a systematic review and network meta-analysis of randomized clinical trials based on regional blocks
Source: Front Pediatr. 2024 Aug 2;12:1417265. doi: 10.3389/fped.2024.1417265 (PMC11327816; doi:10.3389/fped.2024.1417265)
Supplement: Supplementary file 1 [file Datasheet1.pdf]

Supplementary Material

Search terms were used for PubMed

#1 ((((((((((((((((((((("Pediatrics"[Mesh])) OR (children[Title/Abstract])) OR (child[Title/Abstract])) OR (infantile[Title/Abstract])) OR (childre[Title/Abstract])) OR (baby[Title/Abstract])) OR (infants[Title/Abstract])) OR (adolescent[Title/Abstract])) OR (kids[Title/Abstract])) OR (juvenile[Title/Abstract])) OR (childhood[Title/Abstract])) OR (childrens[Title/Abstract])) OR (boy[Title/Abstract])) OR (neonate[Title/Abstract])) OR (infantiles[Title/Abstract])) OR (underage children[Title/Abstract])) OR (girl[Title/Abstract])) OR (infancy[Title/Abstract])) OR (enfant[Title/Abstract])) OR (kid[Title/Abstract])) OR (infanti[Title/Abstract]) 91394

#2 (("Hernia"[Mesh]) OR (Hernias[Title/Abstract])) OR (Enterocele[Title/Abstract]) 86669

#3 ("a"[Mesh]) OR (Analgesias[Title/Abstract]) 48269

#4 (((((randomized controlled trial[Title/Abstract]) OR (Randomized controlled trials[Title/Abstract])) OR (controlled clinical trial[Title/Abstract])) OR (randomized[Title/Abstract])) OR (controlled trial[Title/Abstract])) OR (random[Title/Abstract]) 989284

#5 #1 AND #2 AND #3 AND #4 21

Supplementary Tables

Supplementary Table 1. Network meta-analysis results of pain scores at 2 h

CA						
0.29 (-0.65, 1.33)	II/IHB					
-0.61 (-1.44, 0.22)	-0.90 (-1.96, 0.05)	LAI				
1.73 (-0.07, 3.64)	1.43 (-0.15, 2.97)	2.33 (0.53, 4.28)	PVB			
0.31 (-1.60, 2.24)	0.01 (-1.64, 1.59)	0.91 (-0.93, 2.84)	-1.42 (-3.74, 0.79)	RLB		
0.22 (-0.96, 1.41)	-0.08 (-1.43, 1.24)	0.83 (-0.36, 2.10)	-1.52 (-3.57, 0.55)	-0.08 (-2.24, 2.00)	TAPB	
-0.78 (-2.77, 1.22)	-1.08 (-3.21, 0.98)	-0.18 (-2.18, 1.89)	-2.52 (-5.11, 0.11)	-1.08 (-3.74, 1.53)	-0.98 (-2.66, 0.62)	Control

Supplementary Table 2. Network meta-analysis results of pain scores at 6 h

CA						
-0.11 (-1.64, 1.49)	II/IHB					
0.13 (-0.74, 1.19)	0.24 (-1.25, 1.87)	LAI				
1.23 (-0.92, 3.54)	1.33 (-0.27, 2.95)	1.10 (-1.22, 3.32)	PVB			
0.92 (-1.38, 3.25)	1.04 (-0.72, 2.68)	0.77 (-1.63, 3.07)	-0.29 (-2.64, 2.06)	RLB		
0.99 (-0.77, 2.77)	1.09 (-1.31, 3.46)	0.86 (-1.23, 2.82)	-0.24 (-3.14, 2.63)	0.05 (-2.88, 2.98)	TAPB	
-1.54 (-3.92, 0.97)	-1.45 (-4.30, 1.49)	-1.68 (-4.29, 0.90)	-2.79 (-6.08, 0.72)	-2.47 (-5.75, 0.89)	-2.52 (-4.18, -0.79)	Control

Supplementary Table 3. Network meta-analysis results of pain scores at 24 h

CA		
-0.01 (-2.19, 2.21)	LAI	
0.98 (-0.49, 2.44)	0.99 (-0.67, 2.64)	TAPB

Supplementary Table 4. Network meta-analysis results of rescue analgesia drugs consumption at 2 h

CA		
-0.50 (-1.13, 0.13)	II/IHB	
-0.50 (-1.14, 0.14)	-0.00 (-0.63, 0.63)	LAI

Supplementary Table 5. Network meta-analysis results of rescue analgesia drugs consumption at 6 h

CA			
-0.23 (-2.77, 2.35)	II/IHB		
-0.24 (-2.82, 2.28)	-0.01 (-2.59, 2.58)	LAI	
1.69 (-2.28, 5.84)	1.94 (-2.13, 5.95)	1.96 (-1.23, 5.20)	TAPB

Supplementary Table 6. Network meta-analysis results of rescue analgesia drugs consumption at 24 h

LAI		
33.21 (-8.48, 74.18)	TAPB	
-13.57 (-55.94, 29.09)	-46.76 (-108.36, 13.08)	Control

Supplementary Table 7. Network meta-analysis results of hospital stay

CA		
-0.01 (-12.22, 11.10)	II/IHB	
10.12 (-2.26, 21.53)	9.70 (-6.14, 28.26)	LAI

Supplementary Table 8. Network meta-analysis results of PONV

CA			
1.29 (0.37, 3.85)	LAI		
472425.53 (1.06, 12628403181215706.00)	383612.46 (0.93, 9645668288170744.00)	QLB	
1.33 (0.07, 20.89)	1.02 (0.07, 14.34)	0.00 (0.00, 1.12)	TAPB
